# Recombinant Human Butyrylcholinesterase As a New-Age Bioscavenger Drug: Development of the Expression System

**Published:** 2013

**Authors:** D.G. Ilyushin, O.M. Haertley, T.V. Bobik, O.G. Shamborant, E.A. Surina, V.D. Knorre, P. Masson, I.V. Smirnov, A.G. Gabibov, N.A. Ponomarenko

**Affiliations:** Shemyakin and Ovchinnikov Institute of Bioorganic Chemistry, Russian Academy of Sciences, Miklukho-Maklaya Str., 16/10, Moscow, Russia, 117997; Département de Toxilogie, Centre de Recherches du Servise de Sante des Armées, BP 87, 38702 La Tronche Cedex, France

**Keywords:** bioscavenger, butyrylcholinesterase, CHO cell line, recombinant protein, оrganophosphorus toxins

## Abstract

Butyrylcholinesterase (BChE) is a serine hydrolase (EC 3.1.1.8) which can be found in most animal tissues. This enzyme has a broad spectrum of efficacy against organophosphorus compounds, which makes it a prime candidate for the role of stoichiometric bioscavenger. Development of a new-age DNA-encoded bioscavenger is a vival task. Several transgenic expression systems of human BChE were developed over the past 20 years; however, none of them has been shown to make economic sense or has been approved for administration to humans. In this study, a CHO-based expression system was redesigned, resulting in a significant increase in the production level of functional recombinant human butyrylcholinesterase as compared to the hitherto existing systems. The recombinant enzyme was characterized with Elman and ELISA methods.

## INTRODUCTION

Butyrylcholinesterase (BChE) is a serine hydrolase [EC 3.1.1.8] that has been found in almost all mammalian tissues (in particular, in the lungs, intestine, liver, and blood serum) [1]. The physiological function of BChE has not been determined thus far; however, it is believed to play a key role in maintaining and regulating the activity of neurotransmitter acetylcholine in the central nervous system and neuromuscular endings [2].

BChE can bind stoichiometrically to various acetylcholinesterase- inhibiting toxins. In particular, BChE interacts with organophosphorus compounds, such as sarin, soman, VX and VR gases, as well as with some pesticides. Such data were obtained during experiments on rodents [3] and primates [4]. The animals showed long-term resistance to the action of nerve paralytic agents after intravenous or intramuscular injections of BChE isolated from human serum [5].

Drug therapy methods for treating organophosphorus toxin (OPT) poisoning have been constantly developing for over 60 years. Yet, all of them are far from perfect. Such methods ensure the survival of a patient but cannot help avoid irreversible brain damage and disability. An alternative approach for treatment and prophylaxis of OPT poisoning is the use of bioscavengers [6]. Antibodies, various functional enzymes, and cyclodextrins, which isolate and inactivate highly toxic compounds before they reach their biological targets, can act as bioscavengers [7]. Among all bioscavengers against OPT, only BChE isolated from human plasma has received the status of a “New development drug” from the FDA: in 2006.

Human butyrylcholinesterase is a glycoprotein composed of four identical subunits. Each subunit consists of 574 amino acid residues and 9 polysaccharide chains. The molecular weight of a BChE subunit is 85 kDa, of which 23.9% is attributed to polysaccharide chains [8]. There are several oligomeric forms of BChE: 95% of the BChE in human plasma exists in the tetrameric form; the remaining 5% is represented with trimeric, dimeric, and monomeric forms [9]. The BChE heterodimer with serum albumin is occasionally detected [10]. The oligomeric forms of BChE possess identical, specific activity but differ considerably in terms of their pharmacodynamic characteristics [3].

Human butyrylcholinesterase is nowadays isolated from blood plasma. The purification protocol published in 2005 was designed to process 100 l of human blood plasma in a single cycle [4]. According to experts, the annual supply of blood plasma of the USA has to be processed to obtain 1,000 doses of BChE [11]. Moreover, the use of donor plasma may lead to contamination of the drug with dangerous pathogens.

An alternative method consists in obtaining the recombinant protein. Expression in prokaryotic cells is the simplest (in terms of technology) and the most economically sensible method for producing recombinant proteins. However, attempts to express BChE in* Escherichia coli *have turned out unsuccessful [12].

CHO cells are now widely used to obtain correctly folded and functionally active recombinant products. More than 20 recombinant protein drugs have been produced and approved by the FDA over the past 25 years, including α-glucosidase (Myozyme) [13], the anti-hemophilic factor (ReFacto) [14], coagulation factor IX (BeneFIX) [15], interferon-β ( Avonex) [16], α-galactosidase (Fabrazyme) [17], erythropoietin A (Eprex, Epogen), etc. A number of technical means (such as roller systems, spinners, and bioreactors allowing production of the target protein to an amount of several grams per liter of culture medium) have been developed in order to ensure efficient expression of recombinant proteins in animal cells [18–20]. In addition, expression efficiency is achieved by using strong promoters, such as the elongation factor 1α promoter (EF-1) [21] or the cytomegalovirus (CMV) promoter [22].

In 2002, a recombinant, low-glycosylated BChE was obtained in a nonlymphoid CHO cell line [23]. The yield of the target protein produced in roller bottles was 2–5 mg per liter of the growth medium, whereas production levels need to be at least 50–100 mg/l of the growth medium in order to make economic sense.

Beside increasing the production levels of recombinant BchE, researchers are also focusing on obtaining a product with improved pharmacodynamic properties. Peptides of unknown origin with molecular weights ranging from 2072 to 2878 Da and the overall amino acid sequence PSPPLPPPPPPPPPPPPPPPPPPPPLP have been detected recently as a fragment of human butyrylcholinesterase tetramer. These peptides are believed to play an important role in the formation of the quaternary structure of BChE by binding to the C-terminal domains of its subunits [24]. A proline-rich peptide of N-terminal domain of the collagen-like protein ColQ (PRAD, proline-rich attachment domain) was demonstrated to play an essential role in BChE oligomerisation: the coexpression of peptide PRAD consisting of 45 a.a.r. and the recombinant BChE in CHO cells increases the production of tetrameric BChE isoforms to 70% [25].

In 2007, the American researchers Huang Y.J. *et al.* managed to produce transgenic goats whose milk contained recombinant BChE. It was demonstrated that 1 liter of milk obtained from the transgenic animals contained 1–5 g of active BChE. However, the obtained enzyme was not glycosylated enough, which greatly reduced its pharmacological activity [26].

In 2010, a group of American, Canadian, and Israeli scientists proposed to express recombinant BChE in trasgenic plants [27]. After excessive PEGylation, the pharmacodynamic characteristics of the recombinant enzyme were comparable to those of human plasma BChE. Unfortunately, the clinical use of this drug is complicated as trangenic plants are not allowed by the FDA as a source of recombinant enzymes for therapeutic purposes.

Thus we conclude that, there is no efficient, economically sensible system for the expression of recombinant BChE today. The purpose of this work was to create such an expression system.

## EXPERIMENTAL

**Reagents and materials**

Reagents produced by the following companies were used: Panreac, Amresco and Sigma, (USA); Merck (Germany); DNA plasmid isolation kit, PCR fragment purification kit, Agarose gel DNA extraction kit (Qiagen, USA); restriction enzymes and DNA-modifying enzymes (Fermentas, Lithuania), growth media and components of growth media (Gibco, USA); pcDNA3.1/Hygro, pBudCE 4.1 (Invitrogen, USA), pET 28a (Novogen, USA) vectors. Plasmids pGS / BChE and pRc/RSV-rQ45 were kindly provided by P. Masson (Centre de Recherches du Service de Sante des Armees, Toxicology Department, La Tronche, France) and O. Lockridge (UN MC, Omaha, USA).

**Bacterial strains**

The following *E. coli *strains were used: DH5α, BL21 (DE3) and XL2-Blue (Novagen, USA).

**Cell lines**

A CHO-K1 cell line (Sigma, USA) utilizing the conventional methods for maintaining animal cell lines was used [28]. The cells were grown in culture flasks or plates in a DMEM medium containing 10% fetal bovine serum and 2 mM *L*-glutamine in an incubator at 37°C, 5% CO_2_.

**Fig. 1:**
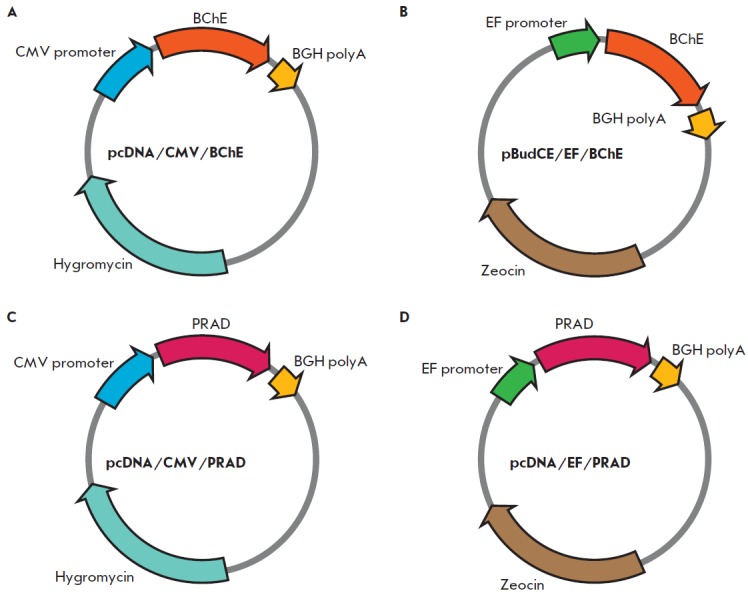
Expression vectors used in this study. (A) pcDNA/CMV/BChE, (B) pBudCE/EF/BChE, (C) pcDNA/CMV/ PRAD, (D) pcDNA/EF/PRAD

**Construction of the expression vectors**

**Table 1:** Oligonucleotide primers used for cloning

Primer 1	TCA AGC CTC AGA CAG TGG TTC
Primer 2	GAA GAA GCT TGT ACA ATA TGC ATA GCA AAG TCA CAA TC
Primer 3	AAG TGG TTC CTT TAA TGC TCC T
Primer 4	ATA TGC GGC CGC TCA TTC TAA GAC ACT TGA TTA TTT CAG T
Primer 5	ATA TGC TAG CGA AGA TGA CAT CAT AAT TGC AAC A
Primer 6	ATA TGC GGC CGC TCA CAG AAA CTT GCC ATC ATA AAC ATG
Primer 7	ATA TGC TAG CGC TCG GGT TGA AAG AGT TAT TGT

1) Construction of the expression vector pcDNA/Hygro/ CMV/BChE (*Fig. 1A*).

Plasmid pGS/BChE carrying the DNA fragment encoding human butyrylcholinesterase was treated with the restriction endonucleases *HindIII *and *ApaI*. A 1914-bp-long DNA fragment was purified by electrophoresis in a 1% agarose gel, followed by elution using the QIAguick Gel Extraction Kit and clonning into the dephosphorylated vector pcDNA3.1/Hygro.

2 ) Construction of the expression vector pBudCE /EF/BChE (*Fig. 1B*).

In order to obtain this construct, the vector pBud- CE 4.1 was modified; the DNA fragment corresponding to the CMV promoter was removed, thus allowing one to construct the vector pBudCE /EF. Plasmid pGS/BChE was treated with restriction endonuclease* BglII*. The required 1832-bp-long DNA fragment was purified as per the procedure described above and cloned into the similarly digested and dephosphorylated vector pBudCE /EF. Positive clones with the correct orientation of the fragments were determined by PCR using primers 1 and 2 (*Table 1*).

3 ) Construction ofthe expression vector pcDNA/CMV/PRAD (*Fig. 1C*).

Plasmid pRc/RSV-rQ45 [29] containing a sequence encoding the PRAD peptide and FLAG epitope was treated with the endonucleases *HindIII *and *XhoI*. A 252-bp-long fragment was purified by electrophoresis in a 10% polyacrylamide gel, followed by electroelution and clonning into the predigested and dephosphorylated vector pcDNA3.1/Hygro.

4) Construction of the expression vector pcDNA/EF/ PRAD (*Fig. 1D*).

Plasmid pBudCE /EF containing the EF promoter was treated with restriction endonuclease BglII. The digested DNA was filled in using DNA polymerase I Large (Klenow) Fragment; the reaction mixture was treated with endonuclease NheI. A 1223-bp-long fragment was purified by electrophoresis in a 1% agarose gel, followed by electroelution. Plasmid pcDNA/CMV/PRAD was treated with restriction endonuclease *HindIII*, filled in using DNA polymerase I Large (Klenow) Fragment, and the reaction mixture was treated with endonuclease SpeI. The vector obtained was purified as per the procedure described above, dephosphorylated and ligated with the previously obtained DNA fragment corresponding to the EF promoter.

5) Construction of the expression vectors pET 28-C, pET 28-N1 and pET 28-N2 (*Fig. 3A*).

The nucleotide sequences encoding the C-terminal fragment of BChE (322 a.a.r.) and two fragments of the N-terminal peptide of BchE–N1 and N2 (133 and 119 a.a.r., respectively) were obtained by PCR . Plasmid pGS/BChE was used as a template. The following primer pairs were used in the reaction: fragment C – primers 3 and 4, fragment N1 – primers 5 and 6, fragment N2 – primers 7 and 8 (*Table 1*). The PCR products C, N1 and N2 were treated with the restriction endonucleases* NheI *and *NotI*, followed by cloning into the pET 28a vector (digested and dephosphorylated) in the same fashion, yielding the expression vectors pET 28-C, pET 28-N1 and pET 28-N2, respectively.

Electrocompetent DH5α or XL2-Blue strain *E. coli* cells were transformed using ligation mixtures. The primary screening of clones from colonies was performed by PCR . The plasmids isolated from positive clones were further characterized by restriction analysis. The correctness of the assembly of expression vectors and constructs was confirmed by Sanger sequencing. Preparation of electrocompetent cells, transformation, and treatment with restriction enzymes, ligation, PCR and DNA electrophoresis were performed in accordance with the standard procedures [30, 31]. The plasmids were isolated according to [32].

**Expression and purification of the recombinant BChE peptides**

BL21(DE3) strain *E. coli *cells were transformed with the vectors pET 28-C, pET 28-N1 or pET 28-N2 by electroporation. The BChE peptides encoded by plasmids contained six histidine residues at the C-terminus, which enabled their isolation using metal-chelate affinity chromatography.

The cells were cultured at 37°C to OD_600_ = 0.6, followed by induction with an isopropylthio-β-Dgalactoside (IPTG) solution added to a concentration of 1mM . Six hours after the induction, the cells were centrifuged at 5000 rpm for 10 min; the precipitate was re-suspended in a buffer containing 50 mM Tris-HCl pH 8.0, 2 mM EDTA, and 0.1% Triton X-100 in 10% of the initial volume.

All the recombinant BChE polypeptides were expressed in the insoluble form. Lysozyme was added to the cell suspension until a final concentration of 0.1 mg/ml, followed by incubation at 30°C for 15 min under constant stirring to obtain a fraction of the inclusion bodies. MgCl_2_ and DNase were then added to the lysate until concentrations of 8 mM, and 0.1 mg/ml, respectively. Cell lysate was centrifuged for 15 min at 13000 rpm. The precipitate containing the insoluble protein fraction was consecutively washed in solutions containing 50 mM Tris-HCl pH 8.0, 150 mM NaCl, 1% Triton X-100, 50 mM Tris-HCl pH 8.0, 150 mM NaCl, 2 M urea and 50 mM Tris-HCl pH 8.0, 150 mM NaCl, 8 M urea. The resulting fractions were analyzed by protein electrophoresis in 15% PAGE under reducing conditions. Polypeptides C and N1 were detected in the fraction containing 2 M urea; polypeptide N2 was detected in the insoluble protein fraction.

The N1 and C polypeptides were then purified by metal-chelate affinity chromatography under denaturing conditions using IMAC Sepharose 6FF resin (GE Healthcare, USA) in accordance with standard manufacturer’s instructions. The eluates were dialyzed against water produced on a mQ installation (Millipore, USA); the precipitate was pelleted by centrifugation and re-suspended in 50% aqueous ethanol to obtain a finely dispersed suspension.

Polypeptide N2 was purified by repeated washing of the insoluble fraction with a solution containing 50 mM Tris-HCl pH 8.0, 150 mM NaCl, 8 M urea, and 1 mM β-mercaptoethanol. The precipitate was dialyzed against water produced on the mQ installation (Millipore, USA), precipitated by centrifugation and resuspended in 50% aqueous ethanol to produce a finely dispersed suspension.

**Immunization of mice using fulllength human plasma BChE**

BALB/c mice were obtained from the Harlan nursery (UK) and kept in the vivarium of the Pushchino Branch of the Institute of Bioorganic Chemistry (Russian Academy of Sciences), under sterile conditions minimizing contact of the immune system with external antigens (Specific Pathogen-Free status). The age of mice ranged from 6 to 8 weeks. Immunization was carried out by administering 100 μg of BChE per mice in a complete Freund’s adjuvant twice at weekly intervals. Booster immunization of mice was performed intraperitoneally 3 days prior to splenocyte collection by administering 50 mg of BChE in phosphate-buffered saline (PBS) per mice.

**Production of mouse monoclonal antibodies**

Monoclonal antibodies were obtained by the standard methods using cell hybridomas and ascites [33, 34]. Monoclonal antibodies were purified by affinity chromatography on resin HiTrap Protein-A (GE Healthcare, USA) according to the manufacturer’s procedure. Biotinylation of the antibodies was performed using NHS-biotin (GE Healthcare, USA), in accordance with the manufacturer’s methodology.

**Immunization of rabbits with recombinant polypeptides BChE**

Immunization of rabbits was carried out in the vivarium of the Institute of Bioorganic Chemistry, Russian Academy of Sciences. The recombinant polypeptides N1 and N2 of BChE were administered subcutaneously as follows: the first injection contained a suspension of the peptide in a complete Freund’s adjuvant, the second injection (after 28 days) contained a suspension of the peptide in an incomplete Freund’s adjuvant, and the third injection (after 14 days) contained a suspension of the peptide in an incomplete Freund’s adjuvant. Each animal was injected with 200 μg of the peptide. Seven days following the immunization, a total of 10 ml of blood was collected from the ear vein of each animal to obtain a blood serum. The antibody titers were determined by indirect ELISA.

**Immunoenzyme assay (ELISA)**

Various ELISA methods using conventional testing protocols were used in the present work [33, 34].

1) Indirect ELISA was used to determine the antibody titer. For this purpose, 96-well plates MaxiSorp (Nunc, USA) were used to adsorb the purified human plasma BChE, and monoclonal antibodies were subsequently introduced to BChE in various dilutions, or human BChE perptides were adsorbed onto plates, followed by the introduction of monoclonal antibodies or polyclonal rabbit sera in various dilutions. The complex was detected using anti-goat antibodies conjugated with horseradish peroxidase.

2) Competitive ELISA was used to search for a pair of monoclonal anti-human BChE antibodies. For this purpose, 96-well plates MaxiSorp (Nunc, USA) were used to adsorb human plasma BChE and were subsequently incubated with monoclonal anti-BChE antibodies in various dilutions in the presence of 10 ng/ml biotinylated monoclonal antibody 4C6D8. The interaction was detected using a streptavidin–HRP conjugate. The starting concentration of the test antibodies was 100 ng/ml.

3) Sandwich ELISA was performed to determine the BChE concentration. The monoclonal antibodies 4C6D8 were adsorbed onto 96-well plates MaxiSorp (Nunc, USA) and incubated with the BChE samples denatured under various conditions (*Fig. 3B*). The interaction was detected using polyclonal anti-N1 BChE rabbit serum (titer 1:1000). Anti-goat antibodies conjugated with horseradish peroxidase were used to detect the reaction.

**Transfection of eukaryotic cells by lipofection**

Prior to the transfection, impurities and salts were removed from the plasmid DNAs, which were preliminarily linearized using the restriction endonuclease PvuI and BglII (in the case of the vector pcDNA/EF/ PRAD). The lipofection was performed using Lipofectamine^™^ Reagent and Plus^™^ Reagent (Invitrogen, USA) according to the manufacturer’s recommendations.

## EXPRESSION OF RECOMBINANT BCHE BY THE CHO CELLS

The cells were cultured in flasks in DMEM containing a 2% fetal bovine serum and 2 mM L-glutamine at 37°C, 5% CO_2_ (25 cm^2^). After the 50–70% monolayer was achieved, the conditioned growth medium was removed and the cells were washed with an equal volume of sterile 1 x PBS, followed by the addition of an equal volume of the protein-free growth medium Peprotech (Peprotech, USA), EX-Cell (Sigma, USA) or ProCHO4 (Lonza, Switzerland). The cells were then incubated in a protein-free growth medium for 5 days at 37°C and 5% CO_2_. Every 24 hours, a sample of the culture medium was taken to determine the content of the active form of butyrylcholinesterase by Ellman’s test.

**Determining the content of the active form of human BChE by Ellman’s test [35]**

The culture liquid (50 μl) was mixed with 100 μl of sterile 1 X PBS, 100 μl of the resulting solution was transferred into a well of the 96-well plate. Butyrylcholinesterase isolated from human plasma (Sigma, USA) was used as a control to build the calibration curve. Prior to taking measurements, a 100 μl solution of 50 μM dithionitrobenzoic acid and 100 μM butyrylthiocholine iodide in 1 X PBS were introduced into the wells containing the samples and controls. The measurements were performed on a TEC AN GEN ios instrument at a wavelength of 405 nm.

**Purification of the recombinant human BuChE from the growth medium**

The growth medium containing CHO cells was centrifuged at 800 *g *for 5 min to remove cells and at 3500 g for 15 min to remove cellular debris. The supernatant was filtered through Millipore HPWH membranes with a pore diameter of 0.22 μm to remove residual impurities. The purified supernatant was ultra concentrated 3 times using Pellicon PLCT K30 membranes (Millipore, USA), followed by dilution with the coating buffer (a 50 mM potassium phosphate buffer, pH 7.2, 1 mM EDTA). The resulting concentrated medium was applied to the Procainamide-Sepharose 4B affinity resin [4] in the recirculation mode at a speed of 0.5 ml/min overnight at +4°C. The recombinant BChE was eluted from the resin using a NaCl gradient (0–500 mM, 15 column volumes) at a flow rate of 0.5 ml/min. The resulting protein fraction was concentrated on Centricon 10 membranes (Millipore) and additionally purified by gel filtration on a Superdex 200 column (GE Healthcare).

**Determination of the content of BChE isoforms by Karnovsky’s method [36]**

Electrophoretic separation of proteins under native conditions was carried out using the standard Laemmli method [31] with minor modifications. An aqueous stock solution with a 29:1 ratio of acrylamide-N,N,N’,N’- methylene-bisacrylamide was used to prepare the gel. The concentrating (upper) 4% gel was prepared in 0.125 M Tris-HCl, pH 6.9. The separating (lower) 8% gel was prepared in 0.125 M Tris-HCl, pH 8.8. Electrophoretic separation was carried out in a buffer containing 50 mM glycine, 5 mM Tris-HCl pH 8.0. The samples were loaded on a buffer containing 10% glycerol, 0.2 M Tris- HCl pH 7.5. Electrophoresis in a concentrating gel was performed at a current of 8–10 mA per gel plate and in the separating gel at 15–20 mA. After the proteins were separated in a nondenaturing polyacrylamide gel, the gel plate was transferred into a solution containing 125 mM NaOH, 125 mM maleic acid, 11.6 mM sodium citrate, 10 mM CuSO_4_, 550 μM potassium hexacyanoferrate (III), and 2 mM butyrylthiocholine iodide. The gel was incubated in the solution on an orbital shaker at room temperature for 3–8 h.

**Determination of the kinetic constants of recombinant BChE by Ellman’s test**

A modified Ellman’s reaction was used to determine the kinetic constants. A known amount of BChE was added into a solution containing 1 mM dithionitrobenzoic acid in a 0.1 M potassium phosphate buffer pH 7.2; the butyrylthiocholine iodide concentration was varied from 10 μM to 1 mM. The number of BChE active sites was determined by titration in diisopropylfluorophosphate (DFP). The reaction was carried out at 25°C; the absorbance was recorded at 412 nm.

## RESULTS AND DISCUSSION

**Development of a recombinant human butyrylcholinesterase expression system**

The aim of our work was to construct an efficient recombinant human BChE expression system. Since BChE is intended for prophylaxis and treatment of OPT exposure, the CHO line cells were selected as well studied and approved by the FDA recombinant protein expression system.

**Fig. 2:**
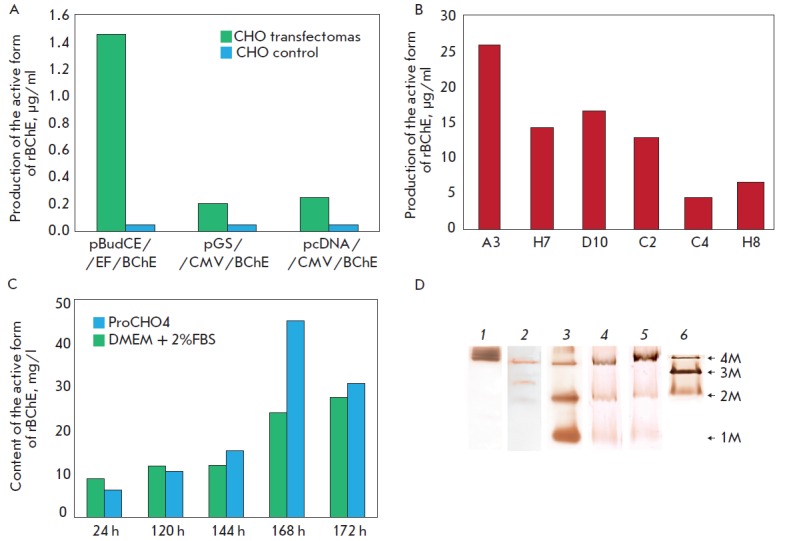
(A) Analysis of the transient transfection of CHO cells with the BChE-containing expression vectors pBudCE/EF/BChE, pGS/CMV/BChE and pcDNA/CMV/BChE. (B) Analysis of recombinant BChE produced by CHO monoclones after stable transfection with a linearized pBudCE/EF/BChE vector. (C) 8% Native PAGE stained by Karnovsky’s method. 1 – Human plasma, 2 – Purified human BChE, 3 – cultural medium of A3 clone, 4 – cultural medium of A3 clone transfected with pcDNA/CMV/PRAD, 5 – clone transfected with pcDNA/EF/PRAD, 6 – cultural medium of A3H9 clone; 4M – tetramer, 3M – trimer, 2M – dimer, 1M – monomer

In order to select a promoter that would provide the most efficient production of BChE, the CHO cell line was transfected via lipofection of the circular plasmid DNA vectors with pGS/BChE [37], pcDNA/CMV/BChE (*Fig. 1A*) and pBudCE /EF/BChE (*Fig. 1B*), which carry the human butyrylcholinesterase gene under the control of various promoters. Samples of the culture media were collected to determine the content of the active form of BChE using Ellman’s test 48 and 72 h following the lipofection. Conditioned medium of the CHO cells line grown under the same conditions as transfectomas were used as the control. The results shown in *Fig. 2A* demonstrate that the expression levels of the vectors pGS/BChE and pcDNA/CMV/BChE were comparable and were equal to approximately 0.2 μg/ml, whereas the expression level of pBudCE /EF/BChE was almost an order of magnitude higher (1.45 μg/ml). Thus, the vector pBudCE /EF/BChE containing the BChE gene under the control of the EF-1 promoter (elongation factor 1) was the most promising construct.

Plasmid DNA of the vector pBudCE /EF/BChE was linearized and transfected via lipofection into the CHO cells in order to obtain stable expression clones. The cells were spread over 24-well plates (1:12) to obtain stable transfectomas 72 h after the lipofection. The selection was performed using Zeocin, which was added to the growth medium at a concentration of 600 μg/ml. After the selection and analysis, the cells were spread over the 96-well plates to obtain monoclones. The production of an active form of BChE at all stages was determined using Ellman’s test. After a comparative analysis of the BChE expression (*Fig. 2B*) the clone A3 was selected for further manipulations. The A3 clone proved a stable producer of recombinant BChE in five genetrations. The next step was to adapt this clone to produce BChE using special protein-free media.

A number of special culture media were tested, including Peprotech (Peprotech), EX-Cell (Sigma), and ProCHO4 (Lonza, Switzerland). In order to adjust the expression conditions, the cells of clone A3 were precultured in a DMEM medium containing 2% fetal bovine serum. After the cells reached a 70–90% monolayer, the medium was replaced with one of the tested proteinfree media, and the cells were incubated for several days. Incubation in Peprotech and EX-Cell media resulted in cell death on the 1^st^ or 2^nd^ day of incubation. These media were deemed unsuitable for this monoclone. During cell incubation in a ProCHO4 medium, a significant increase in rBChE output was observed by the 96^th^ hour of incubation (4 days) (*Fig. 2C*). A decrease in BChE activity on the 5th day can be attributed to the proteolytic activity caused by cell death.

**Fig. 3:**
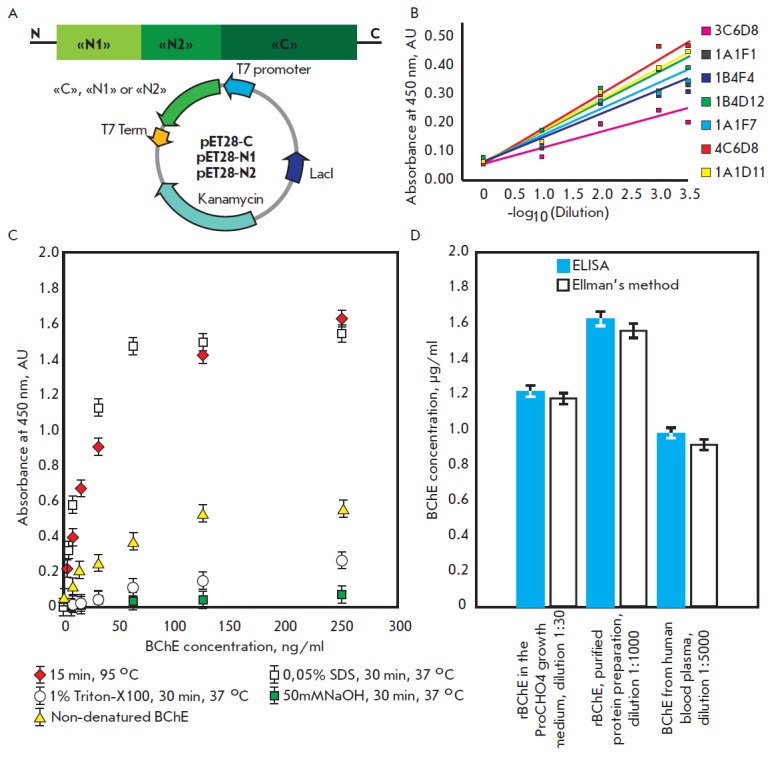
(A) Expression vectors pET28-C, pET28-N1 and pET28-N2 encoding the respective polypeptides of human BChE (B) Competitive ELISA of anti-BChE mouse monoclonal antibodies. The X-axis represents –log_10_(Dilution); starting antibody concentration was 100 ng/ml. All antibodies were tested in pair with the biotinylated 4C6D8 antibody. (C) Determination of the most appropriate method for recombinant BChE denaturation for elaboration of the sandwich ELISA (D) Analysis of the BChE concentration using the Ellman and sandwich ELISA methods gives similar values

The analysis of the oligomeric composition of rBChE produced by clone A3/CHO using Karnovsky’s method (*Fig. 2D, 3*) demonstrated that rBChE was primarily present in the monomeric form, and that the amount of the tetrameric form was minimal. The BChE tetramer is of interest from the pharmacological point of view, since its half-elimination time is 3 to 4 days, while that of the monomer is several hours [4]. It was previously demonstrated that the amount of the tetramerized product increased during the co-expression of the BChE and PRAD peptides (collage-like protein ColQ domain) [37]. Furthermore, addition of a chemically synthesized peptide (a component of BChE) to the growth medium results in tetramerization of the recombinant protein [24]. PRAD peptides and the proline-rich peptide of BChE are very similar in terms of their structure and, therefore, can have similar properties. However, the synthesis of peptides containing tandem proline residues is complicated and characterized by a low yield, and is unprofitable under conditions of biotechnological production. Hence, we decided to use the co-expression of BChE and the PRAD peptide under the control of different promoters. For this purpose the expression vectors pcDNA/ CMV/PRAD (*Fig. 1C*) and pcDN/EF/PRAD (*Fig. 1C*) carrying PRAD under the control of the EF or CMV promoter were transfected into the cells of clone A3 by lipofection. 72 h following the transfection, the presense of the tetrameric form of BChE in the medium was controlled electrophoretically using Karnovsky’s method. The cells of clone A3, which were transfected with plasmid pcDNA3.1/EF/PRAD, were selected based on the results of the analysis (*Fig. 2D, 4, 5*). The use of the EF promoter in this case allows one to obtain cells capable of producing the tetrameric form of BChE in larger quantities. The expression vector pcDNA/EF/PRAD was linearized using restriction endonuclease *BglII *and transfected into the cells of clone A3 by lipofection in order to obtain stable producer clones. The selection was carried out by adding 1.5 mg/ml of hygromycin B and 600 μg/ml of Zeocin to the growth medium. After the selection and analysis, the cells were spread over the 96-well plates to obtain monoclones. Production of BChE isoforms by the monoclones was determined using Karnovsky’s method; the clone A3H9 was selected based on the results. Following the optimization of expression conditions in the ProCHO4 growth medium (Lonza), a stable producer clone, A3H9, characterized by the production of tetrameric and dimeric forms of BChE and complete absence of monomer production was obtained in accordance with the previously described scheme (*Fig. 2D, 6*).

**Development of a system for detecting and assessing the production of the recombinant protein**

In the present work, the evaluation of the efficiency of the transfection and selection of the CHO cell line clones producing recombinant BChE was conducted with respect to the functional activity of the enzyme using Ellman’s [35] and Karnovsky’s [36] methods. These techniques are based on the ability of BChE to hydrolyze butyrylthiocholine, which makes them inapplicable in cases when BChE is inactive or inhibited [38, 39].

It is a well-known fact that high-level expression of recombinant products in eukaryotic cells is sometimes accompanied by a decrease in their specific activity (i.e. production of a certain amount of inactive protein). This can be often attributed to the fact that the system of post-translational modifications of a cell cannot cope with the amount of protein produced; hence, inactive products are formed, which are either misfolded, or contain uncleaved propeptide, or other defects. Such problems can be resolved by co-expression of the product with the required chaperones or the enzymes involved in post-translational modifications [40–44]. Thus, it was critical to measure the specific activity of the enzyme during expression.

Thus, our task was to develop a system of direct assessment of the BChE content in the samples. The system was planned to be used to characterize recombinant BChE, quantitatively detect inactive BChE in the growth medium, and to determine the specific activity of the enzyme during purification. The sandwich – ELISA assay is the simplest and most informative method that can be used to determine the concentration of protein in the samples. The analysis of the commercially available anti-human BChE antibodies demonstrated that pairs of noncompeting monoclonal antibodies which can be used to perform sandwich ELISA have not been produced yet.

The sequences corresponding to the C- and Nterminal fragments of BChE (*Fig. 3A*): C (322 a.a.r.), N1 and N2 (133 and 119 a.a.r.) were produced using the prokaryotic expression system and purified. The monoclonal anti-BChE (full-length human) antibodies 3C6D8, 1A1F1, 1B4F4, 1B4D12, 1A1F7, 4C6D8 and 1A1D11 were obtained using the conventional procedures [33]. Competitive ELISA demonstrated that all the antibodies interacted with the C-terminal region of BChE, while competing with each other (*Fig. 3B*). Western hybridization using BChE fragments confirmed the ELISA results (data not shown). In order to overcome the existing problem, polyclonal rabbit sera were obtained using the BChE recombinant polypeptide fragments N1 and N2 as antigens. Identical titers of antibodies against both N-terminal fragments of BChE were detected in sera using ELISA. However, higher ability to bind to full-length BChE was demonstrated by the anti-N1 antibodies. Despite the high level of specific interaction between the antibodies and BChE during the indirect ELISA, the maximum signal during sandwich ELISA did not exceed 0.6 rel. units. A hypothesis was put forward that partial denaturation of the antigen would increase the availability of the epitopes and thereby increase the sensitivity of the method. Among the tested techniques for denaturing BChE (heating, adding detergents or alkali), incubation at 95°C for 15 min turned out to be the most efficient (*Fig. 3C*). Based on the results of the analysis, the antibody 4C6D8 was selected from a panel of monoclonal antibodies. It showed the highest sensitivity when combined with polyclonal rabbit anti-N1-polypeptide antibodies.

A quantitative method for determining the BChE content in samples of the culture medium, purified preparations, and human plasma was developed. The comparison of the BChE concentration in the samples determined using this method with the results obtained using Ellman’s method demonstrated that over 95% of the BChE expressed by clone A3H9 into the growth medium exhibited enzymatic activity (*Fig. 3D*).

**Isolation and functional analysis of the purified rBChE**

rBChE was purified from the growth medium in order to study its functional activity. The developed purification protocol included such stages as ultrafiltration, concentration, affinity purification, and gel filtration. A sample was selected for each purification stage, was analyzed for content of the active form of BChE by Ellman’s test, and total amount of recombinant enzyme by ELISA. The analysis data are listed in *Table 2*. The final yield of the protein obtained with a purity of 95% (according to the electrophoresis) was about 70%.

**Table 2:** Purification of the recombinant BChE from the growth medium

Extraction phase	General BChE activity, AU	Yield, %	Total amount of BChE, mg	Specific activity, AU/mg
Culture medium	915	100	2.03	451
Culture concentrate	890	97	1.96	454
Column effluent from affinity chromatography	825	90	1.81	456
Gel filtration, 21 min fraction	650	71.5	1.41	461

The kinetic parameters of the purified recombinant BChE were determined using BTC within a concentration range of 10 to 1000 μM at an enzyme concentration of 5 nM. The individual kinetic parameters of the BTC hydrolysis reaction were calculated based on these data (*Table 3*). The comparison of the kinetic constants of rBChE and BChE isolated from human plasma demonstrated that the K_M_ values were identical within the calculation error and were equal to 25 and 23 μM [37], respectively. The constants k_2_ (49200 and 39900 min^-1^, respectively) were only slightly different, which apparently is associated with the methods used to determine the concentration of the active sites of the enzymes under study. It has also been determined that butyrylcholinesterase isolated from human blood plasma is characterized by substrate activation in reactions with compounds of choline series. A similar effect is also observed in the case of the hydrolysis of butyrylcholine iodide of the recombinant BChE at substrate concentrations higher than 500 μM. This allowed to carry out estimation of the constant *K_ss_*and parameter* b*. Parameter b for recombinant BChE did not differed (2.4 ± 0.27) from the standard value of 2.5 ± 0.1 [37] considering the error. Thus, it can be concluded that the obtained recombinant BChE is functionally active, and that the structure of the active site is identical in the natural and recombinant molecules of the enzyme.

## CONCLUSIONS

This study allowed us to construct an efficient system for the expression of active human butyrylcholinesterase in CHO cells. The use of the EF-1 promoter made it possible to significantly increase production of the recombinant protein (from 3–5 to 40 mg/l). The calculated kinetic constants indicate that the active cite of the enzyme is intact. The analysis of the isoforms of rBChE in the growth medium showed that the enzyme is mainly produced in the dimeric and tetrameric forms.

**Table 3:** Kinetic constants of hydrolysis of the recombinant BChE butyrylthiocholine iodide and BChE isolated from human blood plasma

Constant	rBChE	BChE isolated from human plasma [37]
K_M_, μM	25 ± 1	23 ± 2
k_cat_, min^-1^	49200 ± 800	39900 ± 1800
K_ss_, μM	250 ± 30	140 ± 20
b	2.4 ± 0.2	2.5 ± 0.1

The developed ELISA technique allowed us to quantitatively assess the BChE content in samples of these culture medium, the purified enzyme, and in human plasma. The comparison of the BChE concentrations in the samples with those obtained using Ellman’s method demonstrated that over 95% of the BChE expressed by the A3H9 clone was active and that the specific activity of rBChE was not reduced during purification.

The next phase of the work will be focused on further improvement of the pharmacodynamic properties of the recombinant enzyme by chemical modifications, such as PEGylation [45] or sialylation [46].

## Abbreviations

a.a.r.: amino acid residue
BChE: butyrylcholinesterase
PAGE: polyacrylamide gel electrophoresis
OPT: organophosphate toxin
CHO: Chinese hamster ovary cells
DMEM: Dulbecco’s Modified Eagle Medium

## References

[1] Jbilo O., L’Hermite Y., Talesa V., Toutant J.P., Chatonnet A. (1994). Eur. J. Biochem..

[2] Mesulam M. (2002). Neuroscience.

[3] Saxena A., Sun W., Fedorko J.M., Koplovitz I., Doctor B.P. (2011). Biochem. Pharmacol..

[4] Lockridge O., Schopfer L.M., Winger G., Woods J.H. (2005). J. Med. Chem. Biol. Radiol. Def..

[5] Raveh L. (1997). Toxicol. Appl. Pharmacol..

[6] Patrick M., Daniel R. (2009). Acta Naturae.

[7] Masson P., Nachon F., Broomfield C.A., Lenz D.E., Verdier L., Schopfer L.M., Lockridge O. (2008). Chem. Biol. Interact..

[8] Lockridge O., Bartels C.F., Vaughan T.A., Wong C.K., Norton S.E., Johnson L.L. (1987). J. Biol. Chem..

[9] Lenz D.E., Yeung D., Smith J.R., Sweeney R.E., Lumley L.A., Cerasoli D.M. (2007). Toxicology.

[10] Masson P., Carletti E., Nachon F. (2009). Protein Pept. Lett..

[11] Geyer B.C., Kannan L., Cherni I., Woods R.R., Soreq H., Mor T.S. (2010). Plant Biotechnol. J..

[12] Masson P., Steve A., Philippe P.-T., Oksana L. (1992). Multidisciplinary approaches to cholinesterase functions. Expression
and refoldin of functional human butyrylcholinesterase in E. coli. N.Y.: Plenum Press.

[13] Kishnani P.S., Corzo D., Nicolino M., Byrne B., Mandel H., Hwu W.L., Leslie N., Levine J., Spencer C., McDonald M. (2007). Neurology.

[14] Schwartz R.S., Abildgaard C.F., Aledort L.M., Arkin S., Bloom A.L., Brackmann H.H., Brettler D.B., Fukui H., Hilgartner M.W., Inwood M.J. (1990). N. Engl. J. Med..

[15] White G.C., Beebe A., Nielsen B. (1997). Thromb. Haemost..

[16] Jacobs L.D., Cookfair D.L., Rudick R.A., Herndon R.M., Richert J.R., Salazar A.M., Fischer J.S., Goodkin D.E., Granger C.V., Simon J.H. (1996). Ann. Neurol..

[17] Barngrover D. (2002). J. Biotechnol..

[18] Tabuchi H., Sugiyama T., Tanaka S., Tainaka S. (2010). Biotechnol. Bioeng..

[19] Porter A.J., Dickson A.J., Racher A.J. (2010). Biotechnol. Progr..

[20] Strnad J., Brinc M., Spudić V., Jelnikar N., Mirnik L., Carman B., Kravanja Z. (2010). Biotechnol. Progr..

[21] Mizushima S., Nagata S. (1990). Nucl. Acids Res..

[22] Boshart M., Weber F., Jahn G., Dorsch-Häsler K., Fleckenstein B., Schaffner W. (1985). Cell..

[23] Nachon F., Nicolet Y., Viguié N., Masson P., Fontecilla-Camps J.C., Lockridge O. (2002). Eur. J. Biochem..

[24] Li H., Schopfer L.M., Masson P., Lockridge O. (2008). Biochem. J..

[25] Altamirano C.V., Lockridge O. (1999). Biochemistry.

[26] Huang Y.-J., Huang Y., Baldassarre H., Wang B., Lazaris A., Leduc M., Bilodeau A.S., Bellemare A., Côté M., Herskovits P., Touati M., Turcotte C., Valeanu L. (2007). Proc. Natl. Acad. Sci. USA.

[27] Geyer B.C., Kannan L., Garnaud P.-E., Broomfield C.A., Cadieux C.L., Cherni I., Hodgins S.M., Kasten S.A., Kelley K., Kilbourne J., Oliver Z.P., Otto T.C., Puffenberger I. (2010). Proc. Natl. Acad. Sci. USA.

[28] Freshney R.I. (2005). Culture of animal cells. Oxford, N.Y.:
Wiley-Blackwell.

[29] Duysen E.G., Bartels C.F., Lockridge O. (2002). J. Pharmacol. Exp. Ther..

[30] Sambrook J., Russell D.W. (2001). Molecular Cloning. Cold Spring Harbor. N.Y.; Cold Spring Harbor Lab. Press.

[31] Sambrook J., Fritsch E.F., Maniatis T. (1989). Molecular Cloning: A Laboratory Manual. Cold Spring Harbor. N.Y.; Cold Spring Harbor Lab. Press.

[32] Schlesinger N., Baker D.G., Schumacher H.R. (1997). J. Rheumatol.

[33] Harlow E., Harlow E., Lane D. (1988). Antibodies: A Laboratory Manual. Cold Spring Harbor. N.Y.; Cold Spring Harbor Lab. Press.

[34] Ausubel F.M. (2002). Current Protocols.

[35] Ellman G.L., Courtney K.D., Anders V., Feather-Stone R.M. (1961). Biochem. Pharmacol..

[36] Karnovsky M.J., Roots L. (1964). J. Histochem. Cytochem..

[37] Xie W., Altamirano C.V., Bartels C.F., Speirs R.J., Cashman J.R., Lockridge O. (1990). Mol. Pharmacol..

[38] Bartels C.F., Jensen F.S., Lockridge O., Van der Spek A.F., Rubinstein H.M., Lubrano T., La Du B.N. (1992). Am. J. Hum. Genet..

[39] Wang Y., Boeck A.T., Duysen E.G., van Keuren M., Saunders T.L., Lockridge O. (2004). Toxicol. Appl. Pharmacol..

[40] Preininger A., Schlokat U., Mohr G., Himmelspach M., Stichler V., Kyd-Rebenburg A., Plaimauer B., Turecek P.L., Schwarz H.P., Wernhart W., Fischer B.E., Dorner F. (1999). Cytotechnology.

[41] Wajih N., Hutson S.M., Owen J., Wallin R. (2005). J. Biol. Chem..

[42] Jossé L., Smales C.M., Tuite M.F. (2010). Biotechnol. Bioeng..

[43] Meleady P., Henry M., Gammell P., Doolan P., Sinacore M., Melville M., Francullo L., Leonard M., Charlebois T., Clynes M. (2008). Proteomics..

[44] Roncarati R., Seredenina T., Jow B., Jow F., Papini S., Kramer A., Bothmann H., Dunlop J., Terstappen G.S. (2008). Assay Drug Dev. Technol..

[45] Chilukuri N., Sun W., Naik R.S., Parikh K., Tang L., Doctor B.P., Saxena A. (2008). Chem. Biol. Interact..

[46] Jain S., Hreczuk-Hirst D.H., McCormack B., Mital M., Epenetos A., Laing P., Gregoriadis G. (2003). Biochim. Biophys. Acta.

